# Evaluation of Maternal-Fetal Outcomes After Emergency Vaginal Cerclage Performed With Shirodkar-McDonald Combined Modified Technique

**DOI:** 10.14740/jocmr2108w

**Published:** 2015-03-01

**Authors:** Leonarda Ciancimino, Antonio Simone Laganà, Giovanna Imbesi, Benito Chiofalo, Alfredo Mancuso, Onofrio Triolo

**Affiliations:** a Department of Pediatric, Gynecological, Microbiological and Biomedical Sciences, University of Messina, Via C. Valeria 1, 98125 Messina, Italy

**Keywords:** Cervical incompetence, Emergency vaginal cerclage, Maternal-fetal outcomes, McDonald, Shirodkar

## Abstract

**Background:**

Several techniques of emergency vaginal cerclage have been proposed in case of unexpected and abrupt cervical incompetence (CI), in order to prolong the pregnancy as much as possible and to reduce the adverse maternal-fetal outcomes. The aim of our study was to evaluate the effectiveness of emergency cervical cerclage, performed with the combined modified Shirodkar-McDonald technique.

**Methods:**

We selected 12 cases of emergency vaginal cerclage, performed between January 1, 2008 and June 30, 2013. The age of the patients was between 20 and 38 years (mean 29.0 ± standard deviation (SD) 5.69), parity between 0 and 2 (mean 0.7 ± SD 0.65), and gestational age at the time of admission ranged between 17 and 26 weeks (mean 21.0 ± SD 3.44). In all these cases, we used a combined modified Shirodkar-McDonald technique to perform the procedure.

**Results:**

The neonatal survival rate was 83.3%. The cesarean section rate was 16.7%. The average pregnancy prolongation was 89.9 days, higher than that reported for other studies in the literature.

**Conclusions:**

We can assume that the emergency vaginal cerclage performed with the combined modified Shirodkar-McDonald technique is the best option of surgical therapy for the treatment of unexpected and abrupt CI.

## Introduction

During the second trimester of pregnancy, the dilation of the cervix with prolapse of the lower pole of the membranes into the vagina causes, in most of cases, abortion or preterm delivery (PTD). This condition, defined as cervical incompetence (CI), is often characterized by a positive anamnesis for recurrent abortion in the second trimester or PTD at the beginning of the third trimester, subsequent to asymptomatic dilation of the cervix with prolapse of lower pole of the gestational sac into the vagina, even in the absence of an appreciable myometrial contractile activity [1]. In these cases, the prognosis for the fetus is in most cases unfavorable, and therefore is strongly indicated every possible attempt to prolong the pregnancy as much as possible [2]. In 1948, Palmer and La Comme [3] provided an extensive description of this condition, and in the following years several surgical techniques were developed for its treatment either with vaginal approach [4, 5], and, in special in particular cases, also with transabdominal one [6]. Although the cervical cerclage is worldwide performed since many years, data from literature are not completely exhaustive on its real efficacy for the prevention of PTD, except in certain clinical situations [7, 8]. In particular, accumulating evidence suggests that cervical cerclage provides clear and real benefits only in cases identified as CI (three or more previous late miscarriages or PTD). In these cases, the cervical cerclage in the first half of pregnancy has been shown to significantly reduce the incidence of PTD [9, 10]. Moreover, in cases with a significant reduction in the length of the uterine cervix, the neonatal survival rate after successful cervical cerclage is greater than 93%, compared to 23% observed in cases without the procedure [11]. The essential question is represented by the fact that the diagnosis of CI is not still completely delineated, due to the lack of unique and objective diagnostic criteria; therefore, the results of the cervical cerclage procedures are unsatisfactory, since data collected from randomized controlled trials did not show a clear significant improvement of maternal-fetal outcomes [12-14]. From surgical standpoint, the vaginal cerclage procedure is considerably more advantageous than the transabdominal one, because it is shorter, less invasive, less difficult to perform, needs a short-term hospitalization and does not require a cesarean section at the end of pregnancy. From a strictly technical point of view, the passage of the tape within the cervical tissue can be performed in three different ways. The first way provides the passage of the tape through the entire circumference of the cervix, as described by Shirodkar [4]; in the second procedure the tape is placed through a series of small steps around the cervix, like a “tobacco bag” suture, as described by McDonald [5]. The third method provides to place the tape with four passes (four steps) in the thickness of the uterine cervix. While there are no statistically significant differences between vaginal cerclage performed with the Shirodkar or McDonald technique, it has been suggested that the four steps technique can be considered the most valid method. In fact, it is believed that the few but deep passages of the tape in the thickness of the cervical tissue can result in better grip and seal, and, therefore, have a lower tendency to dislocation, a greater resistance to dilation and a reduced risk of lacerations of the cervix in the case of myometrial contractile activity [2, 15]. In the current study, we used a combination of the Shirodkar and McDonald’s ones that in our opinion is faster and simpler to perform and holds better respect to other surgical procedures of cervical cerclage. Regarding the “timing” of cervical cerclage, we can distinguish four different clinical situations: the first is represented by the prophylactic cerclage, performed before conception; the second is the prophylactic cerclage too, but performed during pregnancy; the third is represented by the “emergency” cerclage, performed after a gradual shortening of the uterine cervix identified with vaginal exploration or transvaginal ultrasound; the fourth clinical situation is represented by “emergency” cerclage, characterized by a marked shortening and dilation of the uterine cervix, with prolapse of lower pole of the gestational sac into the vagina. The “emergency” cervical cerclage has been associated with a high risk of chorioamnionitis (about 40%) and rupture of the membranes (around 70%), both during the performing of the procedure and in the next 2 weeks, due to both the direct trauma of the procedure itself and to the contamination of the membranes after contact with the fluor vaginalis subsequent to cervical dilatation [12]. However, it is widely accepted that the monitoring of length and funneling of the uterine cervix cannot be considered, in high-risk cases, a valid and safe alternative to prophylactic cerclage. Some reports, although based on small cohort, seem to indicate that emergency cerclage associated with antibiotic therapy, tocolytic drug (ritodrine, atosiban, and progestagen) and bed rest may represent for the fetus a chance of survival over 96%, compared to 57% observed in cases where the procedure has not been performed [15, 16]. In light of these findings, the aim of our study was to evaluate the maternal-fetal outcomes after cervical cerclage, performed by the combined technique modified Shirodkar-McDonald.

## Methods

This single center, observational case series is based on a retrospective analysis of all cases of cervical cerclage performed at the Department of Pediatric, Gynecological, Microbiological and Biomedical Sciences (University Hospital “G. Martino”, Messina, Italy), from January 1, 2008 to June 30, 2013. We selected and enrolled only cases identified as “emergency” cervical cerclage. The study design is in accordance with the Helsinki Declaration [17], conforms the Committee on Publication Ethics (COPE) guidelines (http://publicationethics.org/) and was approved by the Institutional Review Board (IRB) of the university hospital in which it was carried out. All the design, analysis, interpretation of data, drafting and revisions followed the Strengthening the Reporting of Observational Studies in Epidemiology (STROBE) Statement: guidelines for reporting observational studies [18], available through the EQUATOR (Enhancing the QUAlity and Transparency Of health Research) network (http://www.equator-network.org/). Each enrolled patient was informed in a comprehensive and complete way about techniques that we were going to perform, and signed an informed consent for the two procedures and for the data collection for research purposes. All patients were admitted in our Ob/Gyn emergency unit. After the admission, we performed vaginal examination which identified significant shortening of the uterine cervix, with a dilation equal or greater than 3 cm and prolapse of the lower pole of the amniotic membrane into the vagina. Then, we immediately started administration of tocolytic drugs (ritodrine or atosiban), progestagen and broad spectrum antibiotics. After informing the patient of the clinical condition and risks, and after obtaining informed consent, we performed vaginal cervical cerclage, under general anesthesia. We placed the patient in the Trendelenburg position, and put the vaginal valves in order to see the uterine cervix, to evaluate its dilation and to check the integrity of amniotic membrane. Then, we prepared a regular Foley catheter (Charriere 16), adapted for this purpose by resection of the end a few millimeters above the balloon system in order to preserve its integrity; subsequently, we did endouterine replacement of the lower pole of the membranes above the internal uterine orifice level, according to the technique reported by many authors [19-21]. We tried to place the Mersilene tape as high as possible on the uterine cervix, using a combination of the Shirodkar and McDonald’s techniques, following described. Imagining the profile of the uterine cervix as clock, according to this technique the Mersilene tape crosses four times the cervical tissue: the first time enters at 12 and comes out at 6 o’clock; later on, the same tape enters again at 6 and comes out at 12 o’clock, ready to be tied with the other end. In our case series this technique was performed always by the same operator, in order to limit the intrinsic bias of an operator-dependent procedure. In the days following the surgery, the patient was kept in bed, in slight Trendelenburg position (in order to avoid excessive pressure on the uterine cervix), under tocolytic and broad-spectrum antibiotic infusion. Moreover, at the appropriate gestational age, we administered corticosteroid therapy for fetal lung maturity acceleration and reduction of respiratory distress at birth. In cases with regular course of pregnancy, the cerclage was kept *in situ* and removed at 35 - 36 weeks of gestational age, and subsequently the patient was then discharged waiting the beginning of spontaneous labor. In cases with infectious complications and/or onset or persistence of contractile uterine activity resistant to tocolytic therapy, we proceeded to the timely removal of cerclage, in order to avoid possible tears and consequent series serious bleeding events in the uterine cervix. Data for continuous variables are expressed as mean ± standard deviation (SD), whereas neonatal survival rate and cesarean section rate as percentages.

## Results

During the considered period, we selected and enrolled 12 cases, identified as “emergency” cervical cerclage. The age of the patients ranged between 20 and 38 years (mean 29.0 ± SD 5.69), parity between 0 and 2 (mean 0.7 ± SD 0.65), and gestational age at the time of admission between 17 and 26 weeks (mean 21.0 ± SD 3.44) (Table 1).

**Table 1:** Characteristics of Enrolled Patients

Case no.	Age	Parity				Obstetric history	GA
		TD	PTD	A	LS		
1	31	0	0	1	0	One abortion	23.4
2	32	0	1	0	0	One preterm delivery at 26 weeks	16.2
3	20	0	1	1	0	One preterm delivery at 26 weeks	20.6
4	38	0	0	1	0	One abortion	18.6
5	33	1	0	1	1	One spontaneous delivery at term, one abortion	23.2
6	22	0	0	2	0	Two abortions	24.4
7	32	1	0	1	1	One spontaneous delivery at term, one abortion	25.3
8	22	0	0	0	0	-	23.0
9	33	0	2	0	0	Two preterm deliveries	16.2
10	28	0	0	0	0	Bicornuate uterus	21.0
11	33	1	1	1	1	One spontaneous delivery at term, one preterm delivery, one abortion	16.1
12	24	1	0	1	1	One spontaneous delivery at term, one tubal pregnancy	24.1

GA: gestational age (weeks) at time of cervical cerclage; TD: deliveries at term; PTD: preterm deliveries; A: abortion; LS: living sons.

Two patients were nulliparous and, therefore, without previous adverse obstetric events, but in one of them it had been previously diagnosed bicornuate uterus (defined according to the American Fertility Society classification [22]) by ultrasound. The pregnancy outcomes after the procedure, the type and gestational age of each delivery, and the perinatal outcomes are shown in Table 2.

**Table 2:** Pregnancy Outcomes, Type and Gestational Age of Each Delivery, Perinatal Outcomes After Vaginal Cervical Cerclage Performed With Combined Modified Shirodkar-McDonald Technique

Case no.	GA cerclage	GA delivery	PP	Type of delivery	AI	Neonatal weight (g)	Note
1	23.4	39.1	109	Vaginal	09-10	3.140	AGH
2	16.2	39.2	160	Vaginal	09-10	2.700	AGH
3	20.6	40.0	134	CS	08-07	2.500	AGH
4	18.6	40.0	148	Vaginal	09-10	3.150	AGH
5	23.2	25.0	12	Vaginal	03-06	600	Neonal death after 24 days
6	24.4	34.5	71	Vaginal	07-08	2.450	AGH
7	25.3	40.3	105	Vaginal	10-10	3.500	AGH
8	23.0	30.1	50	Vaginal	09-10	1.450	AGH
9	16.2	37.0	145	CS	09-10	2.900	AGH
10	21.0	23.0	14	Vaginal	04-06	640	Neonal death after 1 day
11	16.2	35.0	131	Vaginal	10-10	2.640	AGH
12	24.1	24.1	0.17 (4 h)	Vaginal	03-07	700	Neonatal intensive care unit

GA: gestational age (weeks) at time of cervical cerclage and of delivery; PP: prolongation of the pregnancy (days); CS: cesarean section; AI: Apgar index; AGH: apparently good health.

The periods between cervical cerclage and the end of the pregnancy ranged between 4 h (0.17 days) and 160 days (Table 3) [15, 23-27].

**Table 3:** Emergency Cervical Cerclage and Prolongation of the Pregnancy: Comparison With Other Studies in Literature

Authors	Year	Cases	PP
Daskalakis et al [15]	2006	29	61.6
Ochi et al [23]	1994	18	35.2
Artmann et al [24]	2001	19	66.0
Ishikawa et al [25]	2003	10	23.9
Althuisius et al [26]	2003	13	54.0
Makino et al [27]	2004	8	32.9
Our case series	2014	12	89.9

PP: prolongation of the pregnancy (days).

In the last case reported in Table 2 (case 12), the patient came to our observation with a uterine cervical shortening of 60-70% and a dilation of approximately 3 cm; a few hours after the surgery, severe uterine contractile activity occurred, refractory to treatment with atosiban and progestagen; therefore, about 4 h later, it became necessary to proceed with the timely removal of cerclage, in order to avoid possible laceration of cervix; soon after there was the rapid expulsion of the fetus, weighing 700 g, with Apgar index 3 - 7, immediately transferred to the neonatal intensive care unit of our hospital. In two patients (cases 5 and 10), the periods between the cerclage and the delivery were respectively 12 and 14 days and the prognosis was poor for both fetuses, born the first at 25 weeks and the second at 23 weeks of gestational age. In three patients there was the onset of uterine contractions and preterm birth occurred, two of them at 35 weeks (case 6 and case 11), the other at 30 weeks (case 8) of gestational age, all with good prognosis for the newborns. In the other six cases, the period between the cervical cerclage procedure and the delivery was higher, with the delivery occurring regularly between 39 and 40 weeks of gestational age. Four of these patients (cases 1, 2, 4 and 7) had a spontaneous delivery, in other two we had to perform cesarean section: one (case 9) for abnormal fetal presentation, the other (case 3) for intrauterine growth restriction and fetal suffering in labor. In all these six cases fetal conditions at birth were quite satisfactory (Apgar index between 8 and 10) and neonatal final prognosis was favorable. Altogether (Table 2) in the 12 patients of our study the average gestational age at the time of cervical cerclage was 20.9 weeks (ranging between 16.2 and 25.3 weeks), neonatal survival rate was 83.3%, and cesarean section rate was only 16.7%. The average pregnancy prolongation was 89.9 ± 58.30 days, higher than that reported for other studies in the literature (Table 3) [15, 23-27].

## Discussion

The identification of unexpected and abrupt CI in the second trimester of pregnancy is a very critical situation, configuring an important risk of adverse maternal-fetal outcome [28]. Numerous literature reports showed that in most cases this condition evolves within a short time, according to the weeks of gestation, in abortion or PTD [25, 29-31]. Since the fetal prognosis is strictly conditioned and dependent by the gestational age, every attempt possible to prolong the pregnancy is mandatory and must be proposed and pursued with the utmost determination. As previously evidenced, the emergency cerclage can be performed by using three vaginal currently standardized techniques in literature: Shirodkar, McDonald, four steps. In our case series we used a Shirodkar-McDonald combined modified technique that in our opinion is faster and simpler to perform and holds better respect to other surgical procedures of cervical cerclage; in fact, our technique results in higher prolongation of the pregnancy than that obtained by other authors (Table 3), without however causing particularly unfavorable maternal-fetal outcomes (Table 3). In addition, the number of cases in our study, although small, is comparable with the case series of other authors (Table 3). In conclusion, we can assume that the emergency vaginal cerclage performed with the combined modified Shirodkar-McDonald technique is a viable option of surgical therapy for the treatment of unexpected and abrupt CI. Nevertheless, there is still the need for future studies, on larger cohorts and with higher statistical power, to verify which of the techniques of vaginal cervical cerclage is more effective in emergency conditions, defining the most appropriate indications in relation to the characteristics of the patient, and considering the variability of maternal and fetal outcomes in the medium to long term on the basis of these parameters.
